# Managing elective surgery without preoperative testing during COVID-19 pandemic – Correspondence

**DOI:** 10.1016/j.amsu.2021.102529

**Published:** 2021-06-29

**Authors:** Petrovic Sabrina, Miklavcic Martina, Bitenc Marko, Beovic Bojana

**Affiliations:** Surgery Bitenc, Ljubljana, Slovenia, Vilharjev Podhod 1, 1000, Ljubljana, Slovenia; University Medical Centre Ljubljana, Zaloška Cesta 7, 1000, Ljubljana, Slovenia

**Keywords:** COVID-19 pandemic, Elective surgery, Transmission

## 1 Introduction

COVID-19 pandemic has a profound impact on surgical healthcare because of suspension of surgeries due to re-location of staff to COVID-19 wards and outbreaks of infections in surgical wards. Testing upon admission was introduced to limit the spread of new coronavirus. However, the reliability of testing is limited by the fact that some patients with negative test may be in the incubation period and may start spreading the virus just after admission. We observed two peaks of COVID-19 epidemic in Slovenia. In the first wave the maximal positivity rate in population was 2,2/100.000 (7-day average). All elective surgeries were suspended for one month with the exemption of cancer surgery. The second wave was much stronger. It peaked at 96,6/100.000 (7-day average) and elective surgeries were performed in limited capacity, mostly with preoperative testing. In this correspondence we describe a successful approach to control the entry of COVID-19 in a specialized surgical hospital throughout the pandemic.

## 2 Methods

Strict COVID-19 protocol was followed in each patient before, during and after surgery. All surgeries were scheduled maximum of 10 days in advance. Patients were told to take maximum precautions against COVID-19. Each patient was called the day before surgery by a registered nurse and interviewed about COVID-19-related symptoms and recent risky contact according to our standardized questionnaire. The same questionnaire was repeated on the admission day together with body temperature measurement. All asymptomatic patients without a history of recent contact with COVID-19 were admitted and surgery was performed. Afterwards all patients were transferred to the surgical department where masks were mandatory and visits were forbidden. When infection with COVID-19 was suspected, PCR test was performed. Patient with positive result was transferred to a COVID-19 ward. All medical staff and patients who had been in contact with positive patients were tested and sent to isolation. If negative, testing was then repeated in 3–5 days or at the onset of any cold-like symptoms.

### 2.1 Results

Between March 16th and May 31st^,^ 2020 and between October 19th^,^ 2020 and February 28th^,^ 2021, 1049 patients were scheduled for surgery among which 18 (1.7%) were cancelled due to symptoms or recent risky contact (Table 1). During the first period one patient (0.3%) developed cough and fever, however the PCR test turned out negative. During the second period 21 (2.9%) patients developed symptoms or signs of COVID-19 among which 1 (0.14%), who had fever and sudden low oxygen saturation, tested positive and was immediately transferred to COVID-19 ward. Out of 6 nurses that had been in contact with positive patient no one developed symptoms of the disease. However, all of them were sent to isolation and in 3 days two of them tested positive.

**Table 1:** Overview of surgeries performed during covid-19 pandemic.

	TYPE OF SURGERY	NUMBER OF SURGERIES	SURGERIES IN 1st PERIOD	SURGERIES IN 2nd PERIOD
TOTAL SCHEDULED		**1049**	334	715
CANCELLED		**18**	9	9
- Due to symptoms		**14**	6	8
- Due to risky contact		**4**	3	1
ONCOLOGIC SURGERY	**Total oncologic**	**210**	**104**	**106**
	Lung resection		71	72
	Prostatectomy		30	25
	Nephrectomy		3	9
OTHER ELECTIVE SURGERY	**Total elective**	**821**	**219**	**602**
	Hernia repair		47	97
	Cholecystectomies		17	59
	Anti-reflux surgery		6	33
	Microdiscectomy or Hemilaminectomy		59	180
	Shoulder surgery		21	35
	Thyroid resection		20	60
	Other thoracic benign surgeries		0	4
	Knee prosthesis		1	17
	Varicose vein surgery		48	117

## 3 Next steps

At the beginning of COVID-19 pandemic, several surgical societies across the world recommended postponement of elective surgery [1]. In our hospital we continued to perform elective surgeries during both epidemiological waves on patients who were not preoperatively tested for SARS-CoV-2, using very strict COVID-19 protocols before, during and after the surgery. The infection was not recognized by the protocol in only one patient who was admitted during the very heavy second wave of epidemic in the country. The approach allowed us to maintain a full programme of elective surgery during the pandemic and some other institution reached similar results as well [2].

PCR testing is the generally accepted tool in preoperative screening of patients. However, viral transmission may occur up to three days before patients may become symptomatic [3]. Therefore, it should be considered that PCR test provides only the information about the infectious status at a certain point of time and that a negatively tested patient might test positive a few days after the initial testing.

It has been demonstrated that elective surgical programs could, based on epidemiological questionnaire in combination with a strict COVID-19 surgical protocol, safely be performed during the ongoing pandemic. Based on our results we certainly do not want to discourage the use of preoperative testing for COVID-19, since it could identify some asymptomatic patients, as described by other authors [4]. However, we would like to underline the high negative predictive value of COVID-19 risk screening questionnaires [5] and that they could help us identify patients who would benefit the most from PCR testing, enabling us to save resources. To develop clearer guidelines on resuming different elective procedures and determine the extent of preoperative screening in times of ongoing COVID-19 pandemic, further studies are necessary.

## Provenance and peer review

Not commissioned, editor reviewed.

## Ethical approval

No Ethical Approval was needed due to the type of study.

## Please state any sources of funding for your research

This research did not receive any specific grant from funding agencies in the public, commercial, or not-for-profit sectors.

## Author contribution

Petrovic Sabrina: Conceptualisation, Investigation, Methodology, Data Analysis, Writing – original draft. Miklavcic Martina: Conceptualisation, Methodology, Data Analysis, Writing – original draft. Beovic Bojana: Conceptualisation, Methodology, Writing – review & editing. Bitenc Marko: Supervising, Writing – review & editing.

## Consent

No patient consent was needed due to the type of study.

## Registration of research studies

1. Name of the registry:/

2. Unique Identifying number or registration ID:/

3. Hyperlink to your specific registration (must be publicly accessible and will be checked):/

## Guarantor

Bitenc Marko, MD, FECTS.

## Declaration of competing interest

All Declarations of interest: none.
